# *Polysiphonia japonica* Extract Attenuates Palmitate-Induced Toxicity and Enhances Insulin Secretion in Pancreatic Beta-Cells

**DOI:** 10.1155/2018/4973851

**Published:** 2018-10-28

**Authors:** Seon-Heui Cha, Hyun-Soo Kim, Yongha Hwang, You-Jin Jeon, Hee-Sook Jun

**Affiliations:** ^1^College of Pharmacy, Gachon University, Incheon 21936, Republic of Korea; ^2^Lee Gil Ya Cancer and Diabetes Institute, Gachon University, Incheon 21936, Republic of Korea; ^3^Gachon Medical and Convergence Institute, Gachon Gil Medical Center, Incheon 21565, Republic of Korea; ^4^Department of Marine Life Science, School of Marine Biomedical Sciences, Jeju National University, Jeju 63243, Republic of Korea

## Abstract

Beta-cell loss is a major cause of the pathogenesis of diabetes. Elevated levels of free fatty acids may contribute to the loss of *β*-cells. Using a transgenic zebrafish, we screened ~50 seaweed crude extracts to identify materials that protect *β*-cells from free fatty acid damage. We found that an extract of the red seaweed *Polysiphonia japonica* (PJE) had a *β*-cell protective effect. We examined the protective effect of PJE on palmitate-induced damage in *β*-cells. PJE was found to preserve cell viability and glucose-induced insulin secretion in a pancreatic *β*-cell line, Ins-1, treated with palmitate. Additionally, PJE prevented palmitate-induced insulin secretion dysfunction in zebrafish embryos and mouse primary islets and improved insulin secretion in *β*-cells against palmitate treatment. These findings suggest that PJE protects pancreatic *β*-cells from palmitate-induced damage. PJE may be a potential therapeutic functional food for diabetes.

## 1. Introduction

It was estimated that 415 million people had diabetes mellitus (DM) in 2015, and this number is projected to increase to 642 million by 2040 (IDF diabetes atlas, 7th edition). DM is a group of chronic metabolic disorders characterized by a deficiency in circulating insulin levels, which results in high blood sugar levels over a prolonged period. Insulin deficiency is caused by a reduction in the number of insulin-producing *β*-cells in both type 1 and type 2 diabetes [1].

In type 2 diabetes, pancreatic *β*-cells are required to secrete increasing amounts of insulin to compensate for increasing insulin resistance. This places *β*-cells under increasing metabolic stress, eventually deteriorating their function and numbers [2–4]. Thus, it is important to preserve the health of *β*-cells. Preventing *β*-cell degeneration is an essential approach for treating DM.

Phytochemicals are regarded as an important source for treating human health problems, including DM. Seaweeds are composed of a variety of bioactive substances such as polysaccharides, pigments, minerals, peptides, and polyphenols, which have valuable pharmaceutical and biomedical potential [5–10]. Numerous studies have demonstrated the beneficial effects of seaweeds for managing DM in animal models of diabetes and human patients [11–16].

*Polysiphonia japonica* is a red seaweed, and some members of its family were shown to have antioxidant [17, 18], antimycobacterial [19], and anticolon cancer [20] activities. However, no studies have examined the effect of *P. japonica* on *β*-cell mass and function. Therefore, in this study, we evaluated whether PJE prevents palmitate-induced *β*-cell dysfunction following exposure to high levels of fatty acids such as those observed in DM.

## 2. Materials and Methods

### 2.1. *Polysiphonia japonica* Extract (PJE)

*Polysiphonia japonica* was collected, rinsed with freshwater to remove the salt, epiphytes, and sand, and stored at −75°C. The frozen samples were lyophilized and finely ground. To prepare the extract, 1 g (dry weight) of the alga was solubilized in 100 mL of 80% methanol for 24 h under continuous shaking at 20°C, and then the extracts were filtered and concentrated under a vacuum in a rotary evaporator (EYELA, Tokyo, Japan) at 40°C.

### 2.2. Cell Culture

The rat pancreatic *β*-cell line Ins-1 was cultured in RPMI 1640 supplemented with 10% fetal bovine serum, 100 U/mL penicillin, 100 *μ*g/mL streptomycin, and 55 *μ*M *β*-mercaptoethanol and was maintained in a humidified incubator with 5% CO_2_.

### 2.3. Isolation of Islets

Islets were isolated from 10-week-old male C57BL/6 mice (Orient Bio, Kyunggi-do, Korea) using the liberase digestion method as described previously [21]. Briefly, after injection of liberase (Roche, Basel, Switzerland) into the bile duct, the swollen pancreas was excised and incubated at 37°C for 20 min. The islets were then separated by Ficoll (Sigma, St. Louis, MO, USA) gradient centrifugation at 2000 × *g* for 10 min. Size-matched healthy islets were hand-picked under a stereomicroscope and maintained in RPMI 1640 containing 5.5 mM glucose supplemented with 10% fetal bovine serum, 100 U/mL penicillin, and 100 *μ*g/mL streptomycin for 24 h.

### 2.4. Assessment of Cell Viability

Cell viability was estimated using a cell counting kit (CCK-8; Dojindo Laboratories, Kumamoto, Japan), which measures mitochondrial dehydrogenase activity. For the CCK-8 assay, Ins-1 cells (5 × 10^4^ cells/well) were seeded into 96-well plates. After 16 h, the cells were incubated with 1 or 2 *μ*g/mL PJE or 0.1, 0.2, 0.4, or 0.8 mM palmitate for 24 h to check the toxicity. To examine the protective effect of PJE, the cells were pretreated with 2 *μ*g/mL PJE for 1 h and then incubated with or without 0.2 mM palmitate (Sigma, St. Louis, MO, USA) for 24 h at 37°C. CCK-8 solution was then added to the wells to a total reaction volume of 110 *μ*L. After 2 h of incubation, the absorbance was measured at a wavelength of 450 nm. The optical density of the formazan generated in the control cells was considered to represent 100% viability.

### 2.5. Measurement of Insulin Secretion

Ins-1 cells (1 × 10^5^ cells/well) or isolated islets (5 or 8 islets) were plated into 24-well plates for insulin secretion measurements as previously described [22]. Briefly, the cells were incubated with KRB buffer containing 3 or 17 mM glucose for 2 h at 37°C. The supernatants were collected, and released insulin was measured using an enzyme-linked immunosorbent assay (ELISA) kit according to the manufacturer's protocol (ALPCO, Salem, NH, USA). Insulin content was normalized to DNA (for islets) or protein (for Ins-1 cells) levels, which was determined using a DC™ protein assay kit (Bio-Rad, Hercules, CA, USA).

### 2.6. Treatment of Zebrafish Embryos with PJE and Palmitate

Transgenic zebrafish expressing enhanced green fluorescent protein under control of the insulin promoter *Tg*(*ins*-egfp) were obtained from Korean Zebrafish Organogenesis Mutant Bank and used in the experiment. Approximately 3 days postfertilization (dpf), embryos (*n* = 6–8) were transferred into a 24-well plate and maintained in 1 mL of embryo media. To determine the effect of PJE on insulin expression, embryos were incubated with or without PJE for 1 day. For palmitate treatment, embryos were incubated in the presence of PJE for 1 h prior to adding palmitate (0.2 mM) for 24 h. Next, the embryos were further incubated with basal glucose (3 mM) or stimulatory glucose (20 mM) for 3.5 h. The embryos were rinsed in embryo media and anesthetized with 2-phenoxyethanol (Sigma) to observe phase and fluorescence images (Leica, Wetzlar, Germany). For confocal microscopy, the embryos were fixed in 4% paraformaldehyde overnight at 4°C and washed with phosphate-buffered saline for 5 min at room temperature. After washing several times with phosphate-buffered saline, the pancreata were isolated from the embryos, stained with DAPI (Invitrogen, Carlsbad, CA, USA) for 5 min, mounted on slides with Vectashield (Vector Laboratories, Burlingame, CA, USA), and observed with a confocal microscope (Zeiss, Oberkochen, Germany). ImageJ software (NIH, Bethesda, MD, USA) was used to quantify the fluorescence and number of cells in the zebrafish. Zebrafish embryos were handled in accordance with the guidelines of Gachon University.

### 2.7. Measurement of Heart Rates

Zebrafish embryos were incubated with 10 *μ*g/mL PJE from 3 to 4 dpf, and heart rates were measured as an indicator of possible PJE toxicity [23]. Counting and recording of atrial and ventricular contractions were performed for 3 min under a microscope, and the results were presented as the average heart rate per minute.

### 2.8. Polyphenol, Carbohydrate, Lipid, and Protein Analysis

To quantify the polyphenol content of PJE, the total phenolic content was estimated using the Folin-Ciocalteu phenol method [24]. The total carbohydrate content of PJE was quantified using the phenol-sulfuric acid method [25]. The lipid content of PJE was determined using a colorimetric sulfo-phospho-vanillin method [26]. The protein concentration of PJE was measured using a DC protein assay kit (Bio-Rad, Hercules, CA).

### 2.9. High-Performance Liquid Chromatography (HPLC) Analysis

Liquid chromatography analysis of PJE was performed on a Waters HPLC system HPLC analysis. A Sunfire C18 ODS 4.6 × 150 mm column (Waters Corporation, Milford, MA, USA) was employed for reverse-phase separations. The mobile phases were 0.1% *v*/*v* formic acid in water and 0.1% *v*/*v* formic acid in acetonitrile at a flow rate of 1 mL/min. The elution gradient for the Sunfire C18 ODS condition was programed as an increasing percentage from 5% to 100% over 60 min, holding at 100% for 10 min, and finally reequilibrating the column at 5% for 10 min. A standard solution containing DMH1 (Tocris, Bristol, UK) was prepared by dissolving DMH1 in distilled water (5 mg/mL). The solution was filtered through a 0.45 *μ*m membrane filter, after which HPLC was performed.

### 2.10. Statistical Analysis

All measurements were carried out in triplicate, and all values are represented as the mean ± S.E. The results were subjected to an analysis of variance with the two-way and Tukey tests to analyze the differences (more than three samples), or Student's *t*-test (two samples) was applied. Values of *p* < 0.05 were considered significant.

## 3. Results

### 3.1. PJE Attenuates Palmitate-Induced Lipotoxicity in Ins-1 Cells

In order to find substances that increase insulin secretion, over 50 seaweed crude extracts were screened. Among them, PJE was the most prominent to insulin secretion.

**Figure 1:**
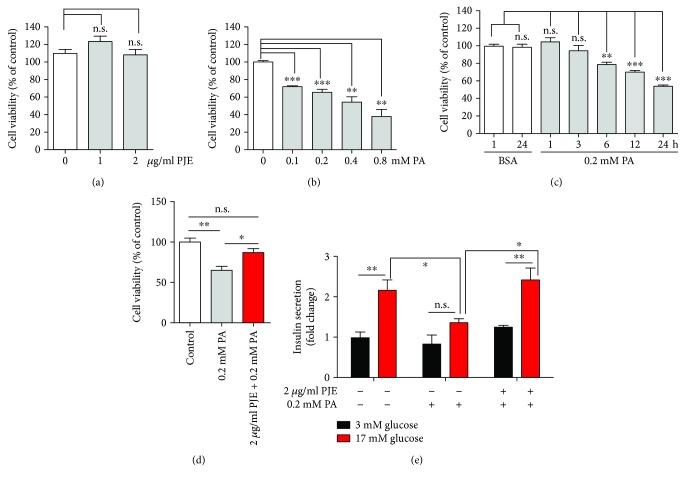
*Polysiphonia japonica* extract (PJE) protects against palmitate-induced lipotoxicity and dysfunction in Ins-1 cells. (a) Ins-1 cells were incubated with the indicated concentrations of PJE for 24 h. (b) Ins-1 cells were incubated with the indicated concentrations of palmitate (PA) for 24 h. (c) Ins-1 cells were incubated with 0.2 mM PA for the indicated times. (d) Ins-1 cells were incubated with 2 *μ*g/mL PJE for 1 h and then further incubated with or without 0.2 mM PA for 24 h. CCK-8 assays were subsequently performed. (e) Ins-1 cells were incubated with 2 *μ*g/mL PJE in 5 mM glucose media for 1 h and then further incubated with or without 0.2 mM palmitate (PA) for 24 h. Thereafter, the cells were starved in 0.2 mM glucose-containing KRB buffer for 2 h. Insulin release was measured after 2 h of incubation in either 3 mM glucose or 17 mM glucose. ELISA assays for insulin were subsequently performed. Data are expressed as the fold change from untreated cells in 3 mM glucose. Experiments were performed in triplicate. ^∗^ *p* < 0.05, ^∗∗^ *p* < 0.01, and ^∗∗∗^ *p* < 0.001. n.s.: no significance.

First, to determine whether PJE protects against palmitate-induced cytotoxicity, Ins-1 cells were treated with either PJE or palmitate alone or were preincubated with PJE for 1 h and then further incubated with palmitate for various doses and times. PJE alone showed no cytotoxicity towards Ins-1 cells in the concentration range tested (1–2 *μ*g/mL) (Figure 1(a)). Significantly lower cell viability was observed in Ins-1 cells treated with palmitate in dose- and time-dependent manners (Figures 1(b) and 1(c)). Pretreatment with 2 *μ*g/mL PJE increased the cell viability to approximately 85% in the presence of 0.2 mM palmitate for 24 h compared to that in the presence of 0.2 mM palmitate alone (Figure 1(d)), indicating that PJE has cytoprotective effects against palmitate-induced damage in Ins-1 cells.

### 3.2. PJE Protects against Palmitate-Induced *β*-Cell Dysfunction in Ins-1 Cells

To investigate whether PJE protects against palmitate-induced *β*-cell dysfunction, we measured insulin secretion from PJE-treated Ins-1 cells in the presence of palmitate. Although palmitate had no effect on basal insulin secretion (3 mM glucose), insulin secretion stimulated by a high glucose concentration (17 mM) was inhibited by treatment with palmitate. When Ins-1 cells were preincubated with 2 *μ*g/mL PJE prior to palmitate treatment, the suppressed insulin secretion was restored to normal levels (Figure 1(e)), suggesting that the PJE has protective effects on the inhibition of insulin secretion in the presence of palmitate in Ins-1 cells.

### 3.3. PJE Promotes Insulin Secretion, but Not *β*-Cell Proliferation, in Zebrafish

**Figure 2:**
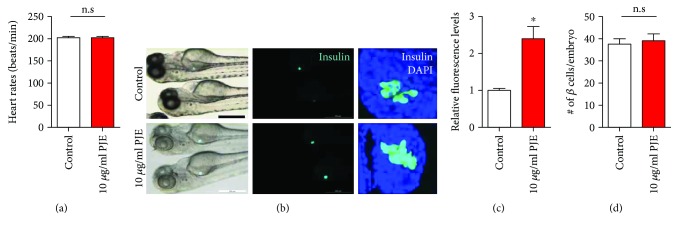
*Polysiphonia japonica* extract (PJE) promotes insulin secretion in zebrafish. Zebrafish were incubated with 10 *μ*g/mL PJE from 3 to 4 days postfertilization. (a) Heart rates of embryos were measured for 3 min. (b) Phase contrast images of zebrafish and fluorescence and confocal microscopy images of the *β*-cell mass of zebrafish. Scale bar: 200 *μ*m. (c) Relative EGFP fluorescence levels from (b). (d) Number of *β*-cells per embryo from (b). *n* = 17–24. ^∗^ *p* < 0.05; n.s.: no significance.

To determine whether PJE directly affects insulin secretion *in vivo*, we used transgenic zebrafish expressing enhanced green fluorescent protein (EGFP) under control of the insulin promoter. Incubation of embryos with 10 *μ*g/mL PJE for 24 h showed no toxic effects, as determined by heart rate measurements (Figure 2(a)). A significantly increased intensity of EGFP was observed in PJE-treated zebrafish embryos (Figures 2(b) and 2(c)). Interestingly, PJE treatment did not alter the number of EGFP-positive *β*-cells (Figure 2(d)). These data suggest that PJE promotes insulin secretion in zebrafish embryos.

### 3.4. PJE Protects against Palmitate-Induced *β*-Cell Dysfunction in Zebrafish

**Figure 3:**
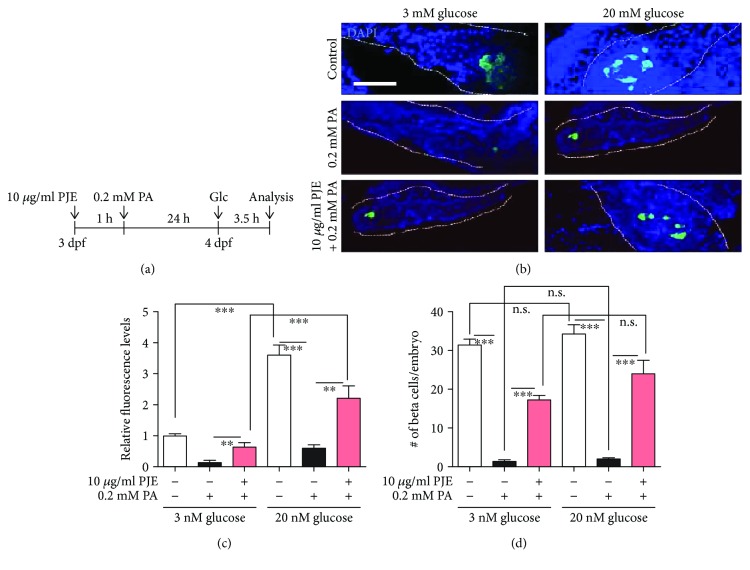
*Polysiphonia japonica* extract (PJE) protects against palmitate-induced *β*-cell dysfunction in zebrafish. (a) Zebrafish were incubated with 10 *μ*g/mL PJE and 0.2 mM palmitate (PA) from 3 to 4 days postfertilization (dpf). PJE was added 1 h prior to PA treatment. Thereafter, the zebrafish incubated with 3 or 20 mM glucose for 3.5 h. (b) Confocal microscopy images of the pancreas of zebrafish. Scale bar: 100 *μ*m. (c) Relative EGFP fluorescence levels from (b). (d) Number of *β*-cells per embryo from (b). *n* = 4–6. ^∗^ *p* < 0.05 and ^∗∗∗^ *p* < 0.001. n.s.: no significance.

Next, we examined whether PJE has a protective effect against palmitate in zebrafish embryos. Embryos were preincubated with 10 *μ*g/mL PJE for 1 h, further incubated with 0.2 mM palmitate for 24 h, and stimulated with 3 or 20 mM glucose for 3.5 h (Figure 3(a)). We found that EGFP-expressing *β*-cells were reduced by palmitate treatment, whereas higher expression of EGFP was observed in PJE-pretreated embryos (Figure 3(b)). Palmitate-treatment decreased both basal insulin secretion (3 mM glucose) and insulin secretion after stimulation with high glucose concentrations (20 mM), while insulin secretion was recovered by PJE pretreatment in zebrafish embryos (Figure 3(c)), suggesting that PJE protects against palmitate-induced insulin secretion dysfunction in zebrafish embryos. Similarly, the number of EGFP-positive (insulin secreting) *β*-cells was reduced by palmitate treatment, and these numbers were recovered by PJE pretreatment under both the 3 and 20 mM glucose conditions (Figure 3(d)).

### 3.5. PJE Protects against Palmitate-Induced *β*-Cell Dysfunction in Mouse Primary Islets

**Figure 4:**
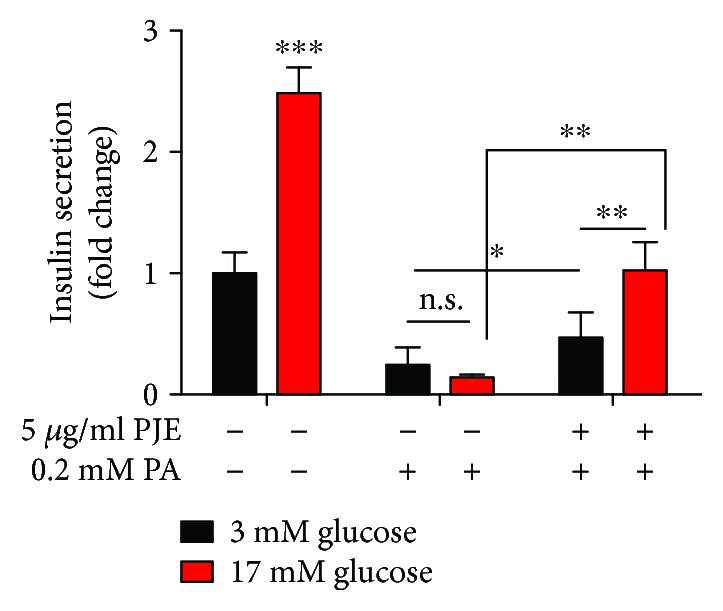
*Polysiphonia japonica* extract (PJE) protects against palmitate-induced *β*-cell dysfunction in mouse primary islets. Islets were incubated with the 5 *μ*g/mL PJE for 1 h and then further incubated with or without 0.2 mM palmitate (PA) for 24 h. Thereafter, the islets were starved in 0.2 mM glucose-containing KRB buffer for 2 h. Insulin release was measured after further incubation with 3 mM glucose or 17 mM glucose by ELISA. Experiments were performed in triplicate. ^∗^ *p* < 0.05, ^∗∗^ *p* < 0.01, and ^∗∗∗^ *p* < 0.001; n.s.: no significance.

As PJE protected against palmitate-induced insulin secretion dysfunction in both the cell line and zebrafish, we next investigated whether PJE protects mouse primary islets. As expected, insulin secretion was decreased by treatment with palmitate, whereas 5 *μ*g/mL PJE pretreatment significantly rescued insulin secretion in palmitate-treated islets (Figure 4). These data suggest that PJE has protective effects on palmitate-induced *β*-cell dysfunction in primary islets.

### 3.6. Chemical Components, Chromatogram, and DMH1 Composition in PJE

**Figure 5:**
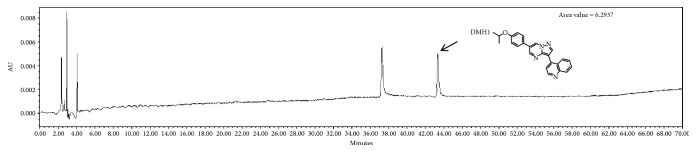
DMH1 was present in *Polysiphonia japonica* extract (PJE).

We determined the levels of chemical components including polyphenol, carbohydrate, lipid, and protein contents of PJE. As shown in Table 1, the proximate composition of PJE was 38.0 ± 2.1 mg/g total phenols, 20.3 ± 1.8 mg/g carbohydrate, 2.9 ± 0.4 mg/g lipid, and 26.5 ± 1.4 mg/g protein. To identify the functional components in PJE, we targeted 4-[6-(4-isopropoxyphenyl)pyrazolo[1,5-a]pyrimidin-3-yl]quinolone (DMH1), a bone morphogenetic protein (BMP) receptor inhibitor [27], as inhibition of BMP has been suggested to affect insulin-secreting *β*-cell growth and function [28]. Thus, we examined whether DMH1 is present in the PJE (5 mg/mL). PJE was evaluated by HPLC, and DMH 1 was separated and eluted at 44 min (Figure 5).

## 4. Discussion

Natural products have been used as alternative treatments for diabetes in many countries [29–34]. Additionally, constituents of seaweeds show antidiabetic potential [35, 36], specifically inhibition of protein tyrosine phosphatase, *α*-glucosidase, and aldose reductase. However, it is unknown whether *P. japonica*, an edible seaweed, can be used to treat diabetic-related diseases at all. In the present study, first, we investigated the protective effects of PJE on palmitate-induced *β*-cell dysfunction.

Increased levels of free fatty acids (FFAs), alone or with hyperglycemia, have been shown to trigger the loss of *β*-cells in both type 1 and type 2 diabetes [37, 38]. In addition, lipotoxicity induced by prolonged elevated FFAs, particularly saturated FFAs such as palmitate, leads to *β*-cell apoptosis and dysfunction [39, 40]. In agreement with previous studies [41, 42], our results showed that exposure to palmitate-induced significant cell death of Ins-1 cells. In addition, palmitate treatment reduced insulin secretion in Ins-1 cells, zebrafish *β*-cells, and isolated mouse islets. In this study, we provide evidence that PJE can prevent *β*-cell death in Ins-1 cells and zebrafish *β*-cells, as well as preserve the dysfunction of insulin secretion both *in vitro* and *in vivo* after exposure to palmitate.

Saturated fatty acids such as palmitate can induce adverse effects, including reduced glucose-stimulated insulin release, suppressed proinsulin biosynthesis, and consequently apoptotic *β*-cell death [39, 43–46]. Several intracellular mediators of fatty acid-induced lipotoxicity have been reported. Palmitate-induced lipotoxicity increases oxidative stress due to intracellular reactive oxygen species accumulation [47–49]. Therefore, PJE may contain components with antioxidative effects, contributing to the prevention of palmitate-induced *β*-cell death and dysfunction. Another possible mechanism is that inhibition of BMP signaling by components in PJE such as DMH1 affects the increase in insulin secretion, as BMP inhibitor is known to improve *β*-cell function [28]. Another possibility is that the increase in or stimulation of glucagon-like peptide-1 receptor by PJE protects against palmitate-induced *β*-cell death and function. Glucagon-like peptide-1 receptor agonists such as exendin-4 are known to prevent palmitate- or H_2_O_2_-induced *β*-cell dysfunction [50, 51]. Further studies are required to clarify the mechanisms for these beneficial effects of PJE on *β*-cell damage.

In conclusion, we found that PJE can effectively protect insulin-secreting *β*-cells from toxicity induced by palmitate. Moreover, PJE improves insulin secretion in Ins-1 cells, zebrafish, and mouse primary islets against palmitate treatment. These results suggest that PJE can be added to functional foods for DM patients and may be useful as a pharmaceutical agent for treating DM.

## Figures and Tables

**Table 1:** Proximate composition of *Polysiphonia japonica* extract (PJE).

Total phenols	Total carbohydrates	Lipid	Protein
38.0 ± 2.1 mg/g	20.3 ± 1.8 mg/g	2.9 ± 0.4 mg/g	26.5 ± 1.4 mg/g

Data are the mean values of triplicate measurements and expressed as the mean ± standard deviation.

## Data Availability

The graphical summary used to support the findings of this study is included in the article.

## Abbreviations

PJE: *Polysiphonia japonica* extract
DM: Diabetes mellitus
FFAs: Free fatty acids
GLP-1: Glucagon-like peptide.

## Supplementary Materials

Supplementary Materials Supplementary figure 1: free fatty acid; palmitate-induced DNA damage consequently impaired insulin secretion; interestingly, pretreatment of *Polysiphonia japonica* extract preserved insulin secretion impairment.
