# Supplementary figures and images for: Polysiphonia japonica Extract Attenuates Palmitate-Induced Toxicity and Enhances Insulin Secretion in Pancreatic Beta-Cells
Source: Oxid Med Cell Longev. 2018 Oct 28;2018:4973851. doi: 10.1155/2018/4973851 (PMC6230388; doi:10.1155/2018/4973851)

## Graphic summary

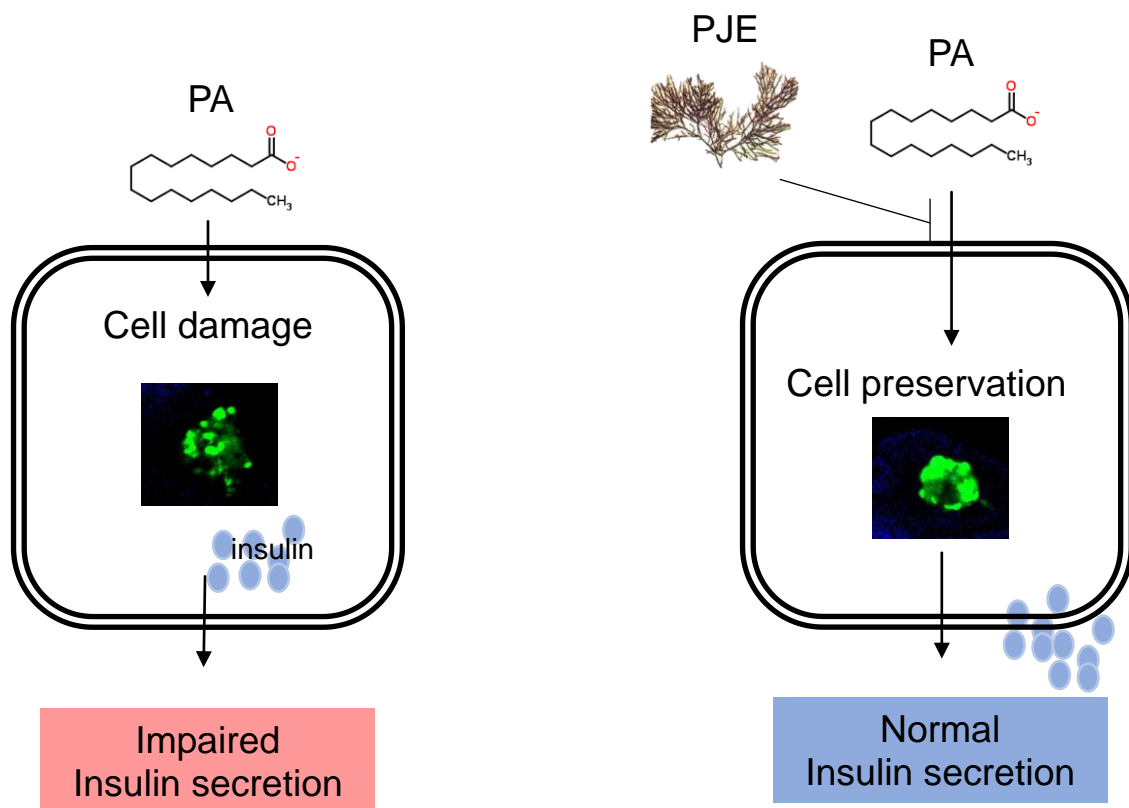

Supplement: Supplementary Materials — Supplementary figure 1: free fatty acid; palmitate-induced DNA damage consequently impaired insulin secretion; interestingly, pretreatment of Polysiphonia japonica extract preserved insulin secretion impairment. [file 4973851.f1.pdf]
